# Association between Blood Heavy Metal Levels and Predicted 10-Year Risk for A First Atherosclerosis Cardiovascular Disease in the General Korean Population

**DOI:** 10.3390/ijerph17062134

**Published:** 2020-03-23

**Authors:** Sungchul Choi, Junhyun Kwon, Pyohyeok Kwon, Changyoon Lee, Sung-In Jang

**Affiliations:** 1 Department of Medicine, Yonsei University College of Medicine, Seoul 03722, Korea; sungchulc@yonsei.ac.kr (S.C.); kkph01@yonsei.ac.kr (P.K.); yoonvly25@yonsei.ac.kr (C.L.); 2 Department of Public Health, Graduate School, Yonsei University, Seoul 03722, Korea; judekwon@yuhs.ac; 3 Institute of Health Services Research, Yonsei University, Seoul 03722, Korea; 4 Department of Preventive Medicine, Yonsei University College of Medicine, Seoul 03722, Korea

**Keywords:** blood cadmium levels, blood lead levels, blood mercury levels, 10-year ASCVD risk

## Abstract

Background: Atherosclerotic cardiovascular disease (ASCVD) is a preventable type of disease, thus, specifying factors that increase the occurrence of this type of disease is needed. Heavy metals such as cadmium (Cd), lead (Pb), and mercury (Hg) have been suggested as possible factors influencing the development of cardiovascular disease. We aimed to link blood heavy metal levels (Cd, Pb, Hg) with 10-year ASCVD risk scores. Methods: A population of 993 men and 1431 women who participated in the Korea National Health and Nutrition Examination Survey (KNHANES) were included. The 2013 American College of Cardiology/American Heart Association (ACC/AHA) pooled cohort equations risk prediction model and Korean Risk Prediction Model (KRPM) were used as means for risk prediction. Following multivariate adjustment; blood Cd; Pb; and Hg levels were divided into quartiles for analysis using linear trends estimation and multiple regression models. Results: There was an overall positive trend between blood Cd, Pb, and Hg levels and 10-year ASCVD risk scores; KRPM risk score increasing by quartile for blood Cd (men *p* < 0.0001, women *p* = 0.0024), Pb (men *p* = 0.0097, women *p* = 0.0330), Hg (men *p* = 0.0096, women *p* = 0.0030) rates and pooled cohort equations risk score increasing by quartile for Cd (men *p* < 0.0001, women *p* = 0.0034) and Hg (men *p* = 0.0099, women *p* = 0.0010) with linear trends. Urban population showed a stronger relationship between blood Cd, Pb, and Hg levels and 10-year ASCVD risk score especially among men with multiple regression analysis. Conclusion: Blood Cd, Pb, and Hg levels are associated with ASCVD risk. Thus, they should be considered while developing preventive measures for ASCVD.

## 1. Introduction

Atherosclerotic cardiovascular disease (ASCVD), which includes nonfatal myocardial infarction, coronary heart disease (CHD) death, and fatal or nonfatal stroke [1], takes up a large proportion of healthcare budgets worldwide [2,3]. In addition to the extensive healthcare expenditures incurred, it is one of the leading causes of death [2,3]. This trend of severity is also true for the South Korean population, where the number of cardiovascular disease patients is high, with one in five deaths occurring due to cardiovascular reasons [4]. Therefore, considering its financial and social burdens, it is critical to estimate the risk of ASCVD and to prevent related risk factors with early measures.

The 10-year ASCVD risk score in the 2013 ACC/AHA Guideline on the Assessment of Cardiovascular Risk is a widely used, reliable estimator of predicted 10-year risk for a first ASCVD event that uses the pooled cohort equation [1]. Individual risk is calculated based on current health status, age, total cholesterol, HDL cholesterol, hypertension treatment, smoking status, and diabetes; in so doing, early initiation of primary prevention can be implemented [5]. This 10-year ASCVD risk score has been reported to have advantages when used in a primary care setting for untreated populations among the Asian population [6]. However, because the 2013 ACC/AHA Guideline on the Assessment of Cardiovascular Risk was created within a specific pool, namely non-Hispanic African-American and non-Hispanic white in the US, numerous studies have reported that this 10-year ASCVD risk score overestimates the risk of ASCVD in Asian populations [6,7,8]. Because of the non-generalizability of this risk score, there have been efforts to create a new pooled cohort equations to fit the Korean population by adjusting the 2013 ACC/AHA pooled cohort equations [7]. The Korean Risk Prediction Model (KRPM) for ASCVD risk was developed as a calibrated version of the 10-year ASCVD risk score for the Korean population, and this novel risk score has been reported to accurately measure the risk of ASCVD among the Korean population [7].

Numerous risk factors have been reported to increase the ASCVD risk or to be associated with increasing the ASCVD risk; among these factors are blood heavy metal levels. Xenobiotic blood heavy metals such as cadmium (Cd), lead (Pb), and mercury (Hg), have been linked with atherosclerotic disease [9]. In particular, Cd and Pb were found to affect aortic atherosclerosis in animal studies [10,11]. There is evidence suggesting that these heavy metals inactivate several enzymes, antioxidants, and amino acids with high affinities for sulfhydryl groups [12], thus possibly having potential to affect atherosclerotic cardiovascular diseases (ASCVD). Emerging evidences are also pointing at the correlative link between heavy metal levels and ASCVD; Cd levels being associated with myocardial infarction and other cardiovascular diseases [13,14], suggestive evidence mounting that support the association between Pb exposure and clinical cardiovascular outcomes [15], Hg having overall vascular effects including oxidative stress, inflammation, and endothelial dysfunction [16], which often is linked with ASCVD [17,18,19]. Taken together, the evidence suggests a need for further scrutiny of the relationship between blood heavy metal levels and ASCVD risk. Therefore, in this study, we investigated the association of blood heavy metal levels of Cd, Pb, and Hg with 10-year ASCVD risk score according to the KRPM with a cross-sectional approach using data from KNHANES.

## 2. Methods

### 2.1. Study Population

Data from the KNHANES, conducted by the Korean Centers for Disease Control and Prevention to evaluate the health and nutritional status of the Korean population, were used for this study. This survey is a national, cross-sectional, population-based survey that assesses the health and nutritional status of the Korean population using health interviews and examinations and nutrition surveys. The nationally representative KNHANES includes approximately 10,000 non-institutionalized civilians as its sample every year [20] and is a reliable dataset which is multi-stage stratified based on 16 metropolitan cities and provinces, as well as administrative divisions and dwelling units. Also, sex, age, residential area ratio, and householder’s education level were used as implicit stratification variables to make the data more representative of the population. Further information about the dataset is available on the website [21] (http://knhanes.cdc.go.kr).

The KNHANES baseline datasets included 8127 and 8150 individuals in 2016 and 2017, respectively. Both 10-year ASCVD risk score used in this study is only applicable to those in the age range of 40 to 80, thus only those in this particular age range was selected as eligible participants (8651 participants). Also, the KNHANES selected one third of its sample for blood heavy metal (Cd, Pb, Hg) levels testing with sub-sampling methods using double-sampling, leaving 3265 participants when exclusion was done from participants within the age range of 40 to 80. Thus, the exclusion criteria were age < 40 years, age > 80 years, participants not tested for blood heavy metal (Cd, Pb, Hg) levels, and missing data for variables. After exclusion of ineligible participants, 2,424 participants, including 993 men and 1431 women, were included in this study. KNHANES survey data for years 2016 and 2017 was exempt from obtaining approval from the Institutional Review Board since the survey was conducted directly by the government of South Korea for public welfare (Bioethics and Safety Act. Republic of Korea). The present study thus did not require additional Institutional Review Board approval because the KNHANES data are publicly available.

### 2.2. Measures

#### 2.2.1. Dependent Variable

This study aimed to investigate the differences in 10-year ASCVD risk score using risk scores from the KRPM for ASCVD and the 2013 ACC/AHA Guideline on the Assessment of Cardiovascular Risk. The 10-year ASCVD risk score is a simple means of assessing the risk of developing a first ASCVD event, including nonfatal myocardial infarction, CHD death, and fatal or nonfatal stroke, over the course of 10 years in a person without any history of nonfatal myocardial infarction (recognized or unrecognized), stroke, heart failure, percutaneous coronary intervention, coronary artery bypass surgery, or atrial fibrillation [1]. The 10-year ASCVD risk score was measured using the equation from the KRPM for ASCVD. The original 10-year ASCVD risk score by 2013 ACC/AHA Guideline on the Assessment of Cardiovascular Risk was also used to supplement these findings. Following the AHA’s recommendation that the equations for non-Hispanic Whites should be used for ethnic groups other than African-Americans, the ASCVD risk score equation for non-Hispanic Whites was used to determine the risk of the target population (Supplementary Tables S1–S3, At the bottom of the article). Both risk scores measure the probability of developing a first ASCVD event in 10 years and are represented as percentages (%) from 0% to 100% as shown in Table 1 and Supplementary Table S1. The 10-year ASCVD risk score was log-adjusted in Table 2 and Table 3 and Supplementary Tables S2 and S3 to meet the requirement of residual normality for accurate linear regression analysis.

#### 2.2.2. Variables of Interest

Blood heavy metal levels were the variables of interest. Blood cadmium (Cd), lead (Pb), and mercury (Hg) levels were measured during the KNHANES 2016 and 2017. Blood Cd and Pb levels were measured using the graphite furnace atomic absorption method on a PerkinElmer AAnalyst AAS-600 (PerkinElmer, Turku, Finland) whereas blood Hg levels were measured using the gold mercury amalgamation method on a DMA-80 (Milestone, Italy). The entire population was divided into four quartiles depending on the blood heavy metal levels: 0%–25%, 25%–50%, 50%–75%, and 75%–100% for each heavy metal.

#### 2.2.3. Covariates

Stratification based on sex was included because the 2013 ACC/AHA Guideline on the Assessment of Cardiovascular Risk has different calculations depending on sex. This study also included age (40–49, 50–59, 60–69, and 70–79 years), income status (low, mid-low, mid-high, and high), job type (white collar, pink collar, blue collar, and unemployed), area of living (rural or urban area), amount of sleep (≥7 h and <7 h), physical activity per week (no activity, walking, moderate-to-vigorous physical activity, and walking and moderate-to-vigorous physical activity (MVPA)). The cutoff point of 7 h was chosen for the amount of sleep according to National Sleep Foundation’s recommendation of sleep duration for young adult and adult groups and older adults group [22]. MVPA included vigorous intensity, moderate intensity, and strength-related physical activities. 

#### 2.2.4. Statistical Analysis

The general characteristics of the study participants were analyzed using analysis of variance (ANOVA). The 10-year ASCVD risk score values were log-transformed for parametric tests. A multiple regression analysis was used to analyze the associations between the blood heavy metal levels and 10-year ASCVD risk score. In addition, as blood heavy metal (Cd, Pb, Hg) levels were divided into quartiles, the linear trend of the variables was evaluated. All our data were stratified by sex to determine whether differences existed between men and women. SAS software, version 9.4 (SAS Institute, Cary, NC, USA) was used for statistical analyses. All *p*-values were two-sided and were considered significant when *p* < 0.05.

## 3. Results 

### 3.1. General Characteristics

General characteristics of the study population are shown in Table 1; the relationships between the KRPM 10-year ASCVD risk score and variables including blood heavy metal levels (Cd, Pb, Hg), age, income status, job type, area of living, sleep duration, and physical activity type are shown stratified according to sex. The 993 male participants had a relatively higher mean 10-year ASCVD risk score of 11.19% (SD 9.99) than the 1,431 female participants, who had a mean risk score of 5.49% (SD 6.10). In men, the following relationships were observed: for blood Cd and Pb levels, there was an observed increase 10-year ASCVD risk score mean with statistically significant difference in mean between quartiles (ANOVA Cd *p* < 0.0001, Pb *p* = 0.0008). The 1431 women showed results similar to that in the men as follows: Blood Cd levels having an observed increase in 10-year ASCVD risk score mean with statistically significant difference in mean between quartiles (ANOVA *p* < 0.0001). Blood Pb levels had a general increasing observed increase, except for the 75% quartile of blood Pb level which did not fit the observed increase of 10-year ASCVD risk score mean, but still having significant statistical mean difference among quartiles (ANOVA *p*-value < 0.0001). For both sex, blood Hg levels did not show a noticeable increasing observed increase but there was a significant statistical mean difference (ANOVA men *p* < 0.0001, women *p* = 0.0387). The 2013 ACC/AHA 10-year ASCVD risk score showed little to no difference in the general characteristics of the study population (Supplementary Table S1).

### 3.2. Association between 10-Year ASCVD Risk Score and Blood Heavy Metal Quartiles

Table 2 shows the linear relationship between the log-adjusted 10-year ASCVD risk score and blood heavy metal quartiles. There was a linear increase for all the three blood heavy metal levels for men: blood Cd (*p* < 0.0001), Pb (*p* = 0.0097), and Hg (*p* = 0.0096) levels showed a linear trend of statistical significance among men. Among women, there was a linear trend for all three heavy metals: Blood Cd (*p* = 0.0024), Pb (*p* = 0.0330), Hg (*p* = 0.0030) levels.

When adjusting for age for multivariate linear regression, job type, area of living, sleep duration, and physical activity, and when comparing the 25% quartile as the reference, Cd’s 50%, 75%, and 100% quartile, Pb’s 100% quartile, and Hg’s 100% quartile had significant statistical meaning for males. Among females, only the 100% quartile for blood Cd levels (b = 0.102 *p* = 0.010) and 100% quartile for Hg levels (b = 0.112 *p* = 0.005) had significant statistical meaning compared to the 25% quartile as reference. All regression analyses were adjusted for age, income status, job type, area of living, sleep duration, and physical activity type per week. There was a difference shown in the Supplementary Table S2 with the original 10-year ASCVD risk that statistically significant linear trend did not appear among Pb for men but others were almost identical.

### 3.3. Subgroup Analysis with Location of Residence and Sleep Duration

Subgroup analyses of the relationship between blood heavy metal quartiles and log-transformed KRPM 10-year ASCVD risk scores by location of residence and sleep duration are shown in Table 3. The urban population exhibited a stronger relationship between 10-year ASCVD risk score and blood heavy metal levels, with Cd and Hg showing significant results especially for men. The rural population showed minor statistically significant numbers. Subgroups by sleep duration showed fewer interesting results in that both less than 7 h and more than 7 h groups did not differ except for the male Cd group 50% quartile, female Pb 100% quartile, and female Hg 100% quartile of under 7 h of sleep showing significant results. As shown in Supplementary Table S3, the 2013 ACC/AHA 10-year ASCVD risk score subgroup analysis had minor differences from the KRPM subgroup analysis.

## 4. Discussion

Blood heavy metal levels are possible influencing factors for cardiovascular diseases, including ASCVD [23,24,25]. The results of this study support this concept by indicating a positive overall trend between blood heavy metal levels (Cd, Pb, Hg) and 10-year ASCVD risk score in the Korean population when adjusted for age, income status, job type, area of living, sleep duration, and physical activity. Specifically, among the three xenobiotic metal levels observed, blood cadmium levels noticeably increased the 10-year ASCVD risk score.

This result is in line with those of previous studies showing that high levels of blood xenobiotic metals (Cd, Pb, Hg) led to increased risk of cardiovascular diseases such as coronal and peripheral arterial disease [26,27], atherosclerosis [28,29,30], and hypertension [11,31]. Our findings extend this concept to cardiovascular disease of atherosclerosis origin. To our knowledge, no other studies suggested the relationship between blood heavy metal levels (Cd, Pb, Hg) and 10-year ASCVD risk score directly. Our findings suggest that ASCVD risk rises with increases in blood heavy metal levels (Cd, Pb, Hg). 

Possible mechanisms have been proposed by earlier studies showing that cadmium acts as an endocrine system disrupter by interacting with various hormonal pathways, often binding to estrogen receptors, mimicking estrogen [32]. Cadmium also increases the formation of oxygen species and binding to metallothionein, thereby interrupting anti-oxidative stress responses [33]. Lead has been shown to increase the production of reactive oxygen species and oxidative tissue damage [34]. Mercury causes oxidative stress by interacting with selenium [35], in addition to its role in inflammation [36]. Many other mechanisms have been suggested; however, no conclusive mechanism has been identified for this linkage between blood heavy metal levels and ASCVD risk. 

One particular focus of this study was the difference of area of residence because cadmium, lead, and mercury are known to be vehicular traffic-related heavy metals [37,38]. Our results indicated that urban populations showed a stronger link between blood heavy metal levels and 10-year ASCVD risk score, whereas rural populations showed no significant associations mainly among men. This result along with possible mechanisms mentioned earlier suggest that area of living influences ASCVD risk, likely because of its relationship with blood heavy metal levels.

Our findings have important real-world implications. It is crucial to reduce heavy metal exposure so as to prevent increase in the incidence of ASCVD. ASCVD is a set of preventable diseases caused by modifiable risk factors [39]. It is important to identify these modifiable risk factors and to take preventive measures to decrease this incidence. Blood heavy metal levels (Cd, Pb, Hg) showed a positive trend when analyzed with 10-year ASCVD risk score in the current study; these factors have been recognized as influencing determinants for other cardiovascular diseases. These data suggest that means to decrease the extent of heavy metal exposures need to be considered to further reduce ASCVD occurrence.

There were some limitations to this study. First, the number of subjects in each subgroup was relatively small. Subgroup analysis with the variables of sleep duration and area of living was performed in the current study, but with a somewhat small number of subjects in each group. There is potential for unintentional bias because of the relatively small number of subjects. Next, covariates were carefully selected to minimize the risk of unexpected errors; however, other possible variables might have influenced the outcome, resulting in unintentional bias. Moreover, the study featured a cross-sectional design, the data is recorded only once for each individual, making it difficult to infer the temporal association between blood heavy metal levels and ASCVD occurrence. In other words, the results could not identify a definite causal relationship and rule out potential bidirectional effects. Lastly, there is a risk of bias due to the complex multi-stage sampling, sub-sampling methods, and missing data.

In conclusion, blood heavy metal levels (Cd, Pb, Hg) showed an overall positive trend with 10-year ASCVD risk score in the Korean population. The urban population had a stronger linkage between blood Cd, Pb, and Hg levels and 10-year ASCVD risk scores. These findings suggest that blood Cd, Pb, and Hg levels are potential influencing factors for ASCVD and suggest that these blood metal levels should be considered in the prevention of ASCVD.

## Figures and Tables

**Table 1:** General characteristics of the study population at the 2016–2017 baseline.

Variables.	Men					Women				
	Subjects		ASCVD ^b^ Risk Score (%) (KRPM ^c^)			Subjects		ASCVD ^b^ Risk Score (%) (KRPM ^c^)		
	N	%	Mean ± SD		*p*-Value *	N	%	Mean ± SD		*p*-Value *
**Total**	993		11.19	9.99		1431		5.49	6.10	
**Quartile Cd**					<0.0001					<0.0001
25%	250	25	8.67	9.76		359	25	4.30	5.81	
50%	249	25	10.75	9.39		357	25	5.42	6.02	
75%	246	25	11.85	9.80		357	25	5.66	5.66	
100%	248	25	13.51	10.39		358	25	6.60	6.67	
**Quartile Pb**					0.0008					<0.0001
25%	249	25	9.40	9.75		358	25	4.28	5.62	
50%	248	25	10.67	10.04		358	25	5.47	6.41	
75%	248	25	11.85	9.73		358	25	5.40	5.56	
100%	248	25	12.84	10.13		357	25	6.82	6.53	
**Quartile Hg**					<0.0001					0.0387
25%	249	25	13.96	11.76		360	25	5.59	6.25	
50%	248	25	10.08	9.40		356	25	6.00	6.94	
75%	248	25	9.80	8.37		359	25	4.73	5.06	
100%	248	25	10.90	9.60		356	25	5.66	5.98	
**Age**					<0.0001					<0.0001
40–49	280	28	2.93	1.94		439	31	1.19	0.66	
50–59	277	28	6.65	3.80		443	31	2.93	1.51	
60–69	243	24	13.37	5.31		340	24	7.35	3.34	
70–79	193	19	26.93	8.32		209	15	16.94	6.46	
**Income status**					0.5946					0.9414
Low	241	24	11.36	9.86		344	24	5.59	6.06	
Mid-low	247	25	11.55	10.84		362	25	5.34	5.86	
Mid-high	239	24	10.42	9.21		339	24	5.58	6.27	
High	266	27	11.39	9.97		386	27	5.48	6.24	
**Job**					<0.0001					<0.0001
White Collar	261	26	5.37	5.31		237	17	1.83	1.82	
Pink Collar	74	7	8.10	6.84		241	17	3.48	3.67	
Blue Collar	406	41	11.21	9.55		308	22	6.11	6.05	
Unemployed	252	25	18.09	10.87		645	45	7.30	7.01	
**Area**					<0.0001					0.0003
Urban	773	78	10.37	9.32		1147	80	5.21	5.90	
Rural	220	22	14.07	11.61		284	20	6.65	6.77	
**Sleep**					0.0025					0.0237
Under 7 h	388	39	9.99	9.07		610	43	5.92	6.64	
Over 7 h	605	61	11.96	10.46		821	57	5.18	5.66	
**Physical Activity**					<0.0001					<0.0001
No activity	149	15	14.11	11.13		227	16	7.23	7.31	
Walking	423	43	11.65	10.29		778	54	5.72	6.09	
MVPA ^a^	47	5	12.05	12.29		35	2	7.90	7.99	
Walking+MVPA	374	38	9.40	8.40		391	27	3.81	4.57	

* ANOVA (one-way) was performed independently for each variable. ^a^ MVPA: Moderate to vigorous physical activity which includes vigorous intensity, moderate intensity, and strength-related physical activities ^b^ ASCVD: Atherosclerotic cardiovascular diseases; ^c^ KRPM: Korean risk prediction model.

**Table 2:** Results of analyzing the effect of the blood heavy metal quartile.

Variables	ASCVD ^b^ Risk Score (Log-Transformed KRPM ^c^)																							
	Men												Women											
	β	e^β **	S.E	*p*-Value	β	e^β **	S.E	*p*-Value	Β	e^β **	S.E	*p*-Value	β	e^β **	S.E	*p*-Value	β	e^β **	S.E	*p*-Value	β	e^β **	S.E	*p*-Value
**Quartile Cd**				<0.0001 *												0.0024 *								
25%	Ref.				-	-	-	-	-	-	-	-	Ref.				-	-	-	-	-	-	-	-
50%	0.132	1.141	0.048	0.006	-	-	-	-	-	-	-	-	0.023	1.023	0.041	0.572	-	-	-	-	-	-	-	-
75%	0.163	1.177	0.053	0.002	-	-	-	-	-	-	-	-	−0.009	0.991	0.037	0.802	-	-	-	-	-	-	-	-
100%	0.269	1.309	0.059	<0.0001	-	-	-	-	-	-	-	-	0.102	1.108	0.039	0.010	-	-	-	-	-	-	-	-
**Quartile Pb**								0.0097 *												0.0330 *				
25%	-	-	-	-	Ref.				-	-	-	-	-	-	-	-	Ref.				-	-	-	-
50%	-	-	-	-	−0.008	0.992	0.057	0.888	-	-	-	-	-	-	-	-	0.036	1.037	0.041	0.381	-	-	-	-
75%	-	-	-	-	0.081	1.084	0.055	0.143	-	-	-	-	-	-	-	-	0.014	1.015	0.038	0.703	-	-	-	-
100%	-	-	-	-	0.117	1.124	0.057	0.041	-	-	-	-	-	-	-	-	0.072	1.075	0.039	0.062	-	-	-	-
**Quartile Hg**												0.0096 *												0.0030 *
25%	-	-	-	-	-	-	-	-	Ref.				-	-	-	-	-	-	-	-	Ref.			
50%	-	-	-	-	-	-	-	-	0.031	1.032	0.053	0.560	-	-	-	-	-	-	-	-	0.024	1.025	0.038	0.525
75%	-	-	-	-	-	-	-	-	0.103	1.109	0.060	0.085	-	-	-	-	-	-	-	-	0.056	1.057	0.043	0.194
100%	-	-	-	-	-	-	-	-	0.126	1.135	0.060	0.037	-	-	-	-	-	-	-	-	0.112	1.118	0.039	0.005
**Age**																								
40–49	Ref.				Ref.				Ref.				Ref.				Ref.				Ref.			
50–59	0.768	2.155	0.054	<0.0001	0.790	2.203	0.056	<0.0001	0.800	2.225	0.055	<0.0001	0.839	2.314	0.035	<0.0001	0.834	2.301	0.035	<0.0001	0.841	2.319	0.035	<0.0001
60–69	1.532	4.629	0.053	<0.0001	1.548	4.704	0.053	<0.0001	1.557	4.746	0.052	<0.0001	1.801	6.055	0.039	<0.0001	1.800	6.052	0.040	<0.0001	1.808	6.097	0.039	<0.0001
70–79	2.199	9.016	0.052	<0.0001	2.218	9.190	0.050	<0.0001	2.251	9.495	0.051	<0.0001	2.594	13.388	0.042	<0.0001	2.597	13.424	0.041	<0.0001	2.612	13.625	0.041	<0.0001
**Income status**																								
Low	Ref.				Ref.				Ref.				Ref.				Ref.				Ref.			
Mid-low	−0.039	0.961	0.056	0.484	−0.044	0.957	0.058	0.451	−0.043	0.958	0.057	0.454	−0.065	0.937	0.045	0.149	−0.067	0.935	0.044	0.131	−0.070	0.932	0.044	0.113
Mid-high	−0.061	0.941	0.064	0.344	−0.080	0.923	0.063	0.204	−0.077	0.926	0.064	0.228	−0.121	0.886	0.044	0.007	−0.119	0.887	0.044	0.007	−0.124	0.883	0.044	0.005
High	0.003	1.004	0.054	0.948	−0.013	0.987	0.053	0.808	−0.043	0.958	0.055	0.429	−0.065	0.937	0.046	0.153	−0.066	0.936	0.046	0.151	−0.078	0.925	0.045	0.084
**Job**																								
White Collar	Ref.				Ref.				Ref.				Ref.				Ref.				Ref.			
Pink Collar	0.146	1.157	0.076	0.055	0.151	1.163	0.081	0.062	0.161	1.174	0.079	0.042	0.086	1.090	0.052	0.097	0.085	1.089	0.052	0.106	0.084	1.087	0.052	0.108
Blue Collar	0.104	1.110	0.053	0.049	0.098	1.103	0.054	0.071	0.124	1.132	0.055	0.023	0.093	1.097	0.051	0.072	0.085	1.089	0.051	0.096	0.090	1.095	0.051	0.079
Unemployed	0.159	1.173	0.057	0.006	0.181	1.198	0.059	0.002	0.200	1.222	0.057	0.001	0.094	1.098	0.042	0.025	0.086	1.090	0.042	0.039	0.094	1.098	0.041	0.022
**Area**																								
Rural	Ref.				Ref.				Ref.				Ref.				Ref.				Ref.			
Urban	−0.021	0.979	0.043	0.623	−0.015	0.985	0.042	0.724	−0.019	0.981	0.042	0.642	−0.053	0.949	0.040	0.183	−0.053	0.949	0.038	0.173	−0.049	0.952	0.038	0.201
**Sleep**																								
Under 7 h	Ref.				Ref.				Ref.				Ref.				Ref.				Ref.			
Over 7 h	−0.033	0.968	0.039	0.406	−0.031	0.970	0.039	0.437	−0.045	0.956	0.040	0.267	−0.055	0.946	0.029	0.058	−0.056	0.945	0.030	0.058	−0.064	0.938	0.029	0.027
**Physical Activity**																								
No activity	Ref.				Ref.				Ref.				Ref.				Ref.				Ref.			
Walking	−0.044	0.957	0.060	0.469	−0.063	0.939	0.059	0.285	−0.056	0.946	0.060	0.350	−0.111	0.895	0.036	0.002	−0.114	0.892	0.036	0.002	−0.117	0.889	0.036	0.001
MVPA ^a^	−0.038	0.963	0.104	0.720	−0.075	0.927	0.107	0.481	−0.082	0.921	0.110	0.455	−0.106	0.900	0.084	0.212	−0.099	0.906	0.085	0.245	−0.115	0.891	0.083	0.166
Walking+MVPA	−0.157	0.854	0.066	0.017	−0.191	0.826	0.065	0.003	−0.193	0.824	0.066	0.004	−0.147	0.863	0.042	0.001	−0.151	0.860	0.042	0.0004	−0.159	0.853	0.042	0.0002

Multiple regression analysis and linear trend results are shown. * *p*-value for linear trend ** e^β shows a percental increase of 10-year ASCVD risk score compared with the reference criteria. ^a^ MVPA: Moderate to vigorous physical activity which includes vigorous intensity, moderate intensity, and strength-related physical activities ^b^ ASCVD: Atherosclerotic cardiovascular diseases ^c^ KRPM: Korean risk prediction model.

**Table 3:** Subgroup analysis of the effect of the blood heavy metal quartile.

Variables			ASCVD ^a^ Risk Score (Log-Transformed KRPM ^b^)															
			Location of Residence								Sleep Duration							
			Urban				Rural				Under 7 h				Over 7 h			
			β	e^β *	S.E	*p*-Value	β	e^β	S.E	*p*-Value	β	e^β	S.E	*p*-value	β	e^β	S.E	*p*-Value
**Men**	**Quartile Cd**	25%	**ref.**				**ref.**				**ref.**				**ref.**			
		50%	0.161	1.175	0.054	0.003	−0.094	0.910	0.101	0.353	0.117	1.124	0.058	0.045	0.151	1.163	0.089	0.090
		75%	0.188	1.207	0.058	0.001	−0.014	0.987	0.107	0.900	0.158	1.171	0.068	0.022	0.196	1.216	0.076	0.011
		100%	0.270	1.310	0.066	<0.0001	0.155	1.168	0.115	0.180	0.232	1.261	0.070	0.001	0.310	1.363	0.088	0.001
	**Quartile Pb**	25%	**ref.**				**ref.**				**ref.**				**ref.**			
		50%	−0.001	0.999	0.064	0.989	0.051	1.053	0.132	0.697	0.010	1.010	0.074	0.897	−0.015	0.985	0.093	0.873
		75%	0.082	1.085	0.057	0.154	0.134	1.143	0.152	0.378	0.029	1.029	0.075	0.702	0.145	1.156	0.081	0.074
		100%	0.133	1.142	0.062	0.032	0.079	1.082	0.119	0.509	0.097	1.102	0.066	0.141	0.183	1.201	0.094	0.053
	**Quartile Hg**	25%	**ref.**				**ref.**				**ref.**				**ref.**			
		50%	0.043	1.044	0.058	0.457	−0.088	0.916	0.100	0.380	0.095	1.100	0.075	0.202	−0.047	0.954	0.082	0.566
		75%	0.095	1.100	0.066	0.152	0.053	1.054	0.115	0.645	0.159	1.173	0.070	0.023	0.010	1.010	0.094	0.919
		100%	0.130	1.139	0.066	0.049	0.028	1.028	0.112	0.803	0.143	1.153	0.074	0.055	0.093	1.097	0.090	0.306
**Women**	**Quartile Cd**	25%	**ref.**				**ref.**				**ref.**				**ref.**			
		50%	0.071	1.074	0.042	0.094	−0.253	0.776	0.100	0.012	0.079	1.082	0.052	0.127	−0.055	0.946	0.065	0.398
		75%	−0.005	0.995	0.040	0.902	−0.006	0.994	0.081	0.937	0.027	1.027	0.047	0.565	−0.074	0.929	0.061	0.226
		100%	0.114	1.121	0.042	0.007	0.051	1.053	0.096	0.591	0.102	1.107	0.055	0.066	0.091	1.095	0.055	0.101
	**Quartile Pb**	25%	**ref.**				**ref.**				**ref.**				**ref.**			
		50%	0.033	1.033	0.046	0.475	−0.001	0.999	0.078	0.986	0.076	1.079	0.050	0.130	−0.020	0.981	0.060	0.744
		75%	0.007	1.007	0.042	0.875	0.003	1.003	0.088	0.976	0.078	1.081	0.047	0.096	−0.068	0.935	0.060	0.259
		100%	0.038	1.039	0.043	0.377	0.212	1.236	0.085	0.013	0.110	1.116	0.048	0.023	0.021	1.021	0.059	0.719
	**Quartile Hg**	25%	**ref.**				**ref.**				**ref.**				**ref.**			
		50%	0.026	1.026	0.043	0.551	0.022	1.023	0.069	0.745	0.053	1.055	0.050	0.286	−0.002	0.998	0.058	0.967
		75%	0.050	1.051	0.046	0.284	0.103	1.109	0.109	0.342	0.067	1.069	0.052	0.203	0.049	1.050	0.066	0.455
		100%	0.113	1.120	0.043	0.009	0.120	1.128	0.089	0.177	0.116	1.123	0.052	0.027	0.110	1.117	0.065	0.090

Mltiple regression analysis results are shown. * e^β shows a percental increase of 10-year ASCVD risk score compared with the reference criteria. ^a^ ASCVD: Atherosclerotic cardiovascular diseases. ^b^ KRPM: Korean risk prediction model

## Supplementary Materials

The following are available online at https://www.mdpi.com/1660-4601/17/6/2134/s1, Table S1. General characteristics of the study population at the 2016-2017 baseline. Table S2. Results of analyzing the effect of the blood heavy metal quartile. Table S3. Subgroup analysis of the effect of the blood heavy metal quartile.

## Abbreviations

ASCVD	Atherosclerotic cardiovascular disease
ANOVA	Analyzed using analysis of variance
CHD	Coronary heart disease
KNHANES	Korea National Health and Nutrition Examination Survey
KRPM	Korean Risk Prediction Model
