# Supplementary material for: Association between Blood Heavy Metal Levels and Predicted 10-Year Risk for A First Atherosclerosis Cardiovascular Disease in the General Korean Population
Source: Int J Environ Res Public Health. 2020 Mar 23;17(6):2134. doi: 10.3390/ijerph17062134 (PMC7142968; doi:10.3390/ijerph17062134)
Supplement: Supplementary file 1 [file ijerph-17-02134-s001.pdf]

Table 1. General characteristics of the study population at the 2016-2017 baseline

Variables	Men					Women				
	Subjects		ASCVD[b] risk score (%)			Subjects		ASCVD[b] risk score (%)		
	N	%	Mean±SD		P-value*	N	%	Mean±SD		P-value*
**Total**	993		17.11	14.35		1,431		7.3	10.42	
**Quartile Cd**					<.0001					0.0002
25%	250	25	13.06	14.03		359	25	5.62	10.00	
50%	249	25	16.70	14.06		357	25	7.18	10.24	
75%	246	25	18.30	14.28		357	25	7.36	9.61	
100%	248	25	20.43	14.10		358	25	9.06	11.50	
**Quartile Pb**					0.0007					<.0001
25%	249	25	14.40	13.71		358	25	5.62	9.50	
50%	248	25	16.57	14.71		358	25	7.25	10.88	
75%	248	25	17.98	14.19		358	25	7.03	9.72	
100%	248	25	19.50	14.38		357	25	9.33	11.20	
**Quartile Hg**					0.0003					0.0295
25%	249	25	20.37	16.22		360	25	7.47	10.59	
50%	248	25	15.48	13.67		356	25	8.25	12.06	
75%	248	25	15.59	12.63		359	25	5.98	8.40	
100%	248	25	17.00	14.18		356	25	7.52	10.23	
**Age**					<.0001					<.0001
40-49	280	28	5.34	4.72		439	31	1.00	0.97	
50-59	277	28	11.59	7.97		443	31	2.93	2.46	
60-69	243	24	20.97	9.59		340	24	9.11	5.59	
70-79	193	19	37.25	12.35		209	15	26.89	12.87	
**Income status**					0.4034					0.8907
Low	241	24	17.73	14.28		344	24	7.49	10.26	
Mid-low	247	25	17.63	15.56		362	25	6.93	9.70	
Mid-high	239	24	15.76	13.29		339	24	7.41	10.64	
High	266	27	17.28	14.17		386	27	7.40	11.03	
**Job**					<.0001					<.0001
White Collar	261	26	8.79	8.86		237	17	1.77	2.76	
Pink Collar	74	7	12.96	9.96		241	17	3.87	5.69	
Blue Collar	406	41	17.54	13.94		308	22	8.00	10.19	
Unemployed	252	25	26.26	15.14		645	45	10.29	12.33	
**Area**					<.0001					0.0007
Urban	773	78	16.04	13.54		1147	80	6.84	10.03	

Rural	220	22	20.88	16.38		284	20	9.18	11.72	
**Sleep**					0.0096					0.0046
<7 hr	388	39	15.64	13.35		610	43	8.21	11.71	
> 7 hr	605	61	18.06	14.89		821	57	6.63	9.30	
**Physical Activity**					<.0001					<.0001
No activity	149	15	21.28	15.42		227	16	10.31	12.83	
Walking	423	43	17.73	14.49		778	54	7.65	10.38	
MVPA[a]	47	5	18.43	18.26		35	2	10.93	13.48	
Walking+MVPA[a]	374	38	14.58	12.69		391	27	4.55	7.63	

[*] ANOVA (one-way) was performed independently for each variable

[a] MVPA: moderate to vigorous physical activity which includes vigorous intensity, moderate intensity, and strength-related physical activities

[b] ASCVD: atherosclerotic cardiovascular diseases

Table 2. Results of analyzing the effect of the blood heavy metal quartile

Variables	ASCVD[b] risk score																							
	Men												Women											
	β	e^β**	S.E	P-value	β	e^β**	S.E	P-value	β	e^β**	S.E	P-value	β	e^β**	S.E	P-value	β	e^β**	S.E	P-value	β	e^β**	S.E	P-value
**Quartile Cd**				<0.0001*												0.0034*								
25%	Ref.				-	-	-	-	-	-	-	-	Ref.				-	-	-	-	-	-	-	-
50%	0.227	1.254	0.063	0.0004	-	-	-	-	-	-	-	-	0.036	1.037	0.060	0.548	-	-	-	-	-	-	-	-
75%	0.270	1.311	0.069	0.0001	-	-	-	-	-	-	-	-	- 0.021	0.979	0.055	0.705	-	-	-	-	-	-	-	-
100%	0.412	1.510	0.073	<.0001	-	-	-	-	-	-	-	-	0.141	1.152	0.058	0.015	-	-	-	-	-	-	-	-
**Quartile Pb**								0.0347*												0.0519*				
25%	-	-	-	-	Ref.				-	-	-	-	-	-	-	-	Ref.				-	-	-	-
50%	-	-	-	-	0.017	1.018	0.077	0.821	-	-	-	-	-	-	-	-	0.011	1.011	0.061	0.859	-	-	-	-
75%	-	-	-	-	0.083	1.087	0.075	0.270	-	-	-	-	-	-	-	-	0.008	1.008	0.055	0.885	-	-	-	-
100%	-	-	-	-	0.139	1.149	0.076	0.069	-	-	-	-	-	-	-	-	0.085	1.089	0.059	0.151	-	-	-	-
**Quartile Hg**												0.0099*												0.0010*
25%	-	-	-	-	-	-	-	-	Ref.				-	-	-	-	-	-	-	-	Ref.			
50%	-	-	-	-	-	-	-	-	0.064	1.066	0.072	0.375	-	-	-	-	-	-	-	-	0.034	1.034	0.056	0.547
75%	-	-	-	-	-	-	-	-	0.166	1.180	0.077	0.032	-	-	-	-	-	-	-	-	0.094	1.098	0.061	0.125
100%	-	-	-	-	-	-	-	-	0.161	1.175	0.083	0.052	-	-	-	-	-	-	-	-	0.171	1.187	0.057	0.003
**Age**																								
40-49	Ref.				Ref.				Ref.				Ref.				Ref.				Ref.			
50-59	0.770	2.159	0.069	<.0001	0.809	2.245	0.072	<.0001	0.821	2.274	0.070	<.0001	1.055	2.871	0.052	<.0001	1.048	2.852	0.052	<.0001	1.056	2.876	0.052	<.0001
60-69	1.458	4.296	0.067	<.0001	1.489	4.434	0.068	<.0001	1.500	4.483	0.066	<.0001	2.276	9.736	0.059	<.0001	2.276	9.738	0.060	<.0001	2.284	9.820	0.059	<.0001
70-79	2.019	7.531	0.067	<.0001	2.058	7.829	0.067	<.0001	2.101	8.172	0.068	<.0001	3.310	27.386	0.065	<.0001	3.315	27.515	0.064	<.0001	3.336	28.097	0.064	<.0001
**Income status**																								

Low	Ref.				Ref.				Ref.				Ref.				Ref.				Ref.			
Mid-low	-0.068	0.934	0.075	0.367	-0.071	0.932	0.076	0.355	-0.068	0.934	0.075	0.363	-0.090	0.914	0.063	0.156	-0.094	0.911	0.063	0.138	-0.098	0.906	0.063	0.119
Mid-high	-0.091	0.913	0.080	0.255	-0.114	0.892	0.079	0.147	-0.114	0.893	0.080	0.157	-0.198	0.821	0.063	0.002	-0.196	0.822	0.062	0.002	-0.203	0.817	0.062	0.001
High	0.008	1.008	0.069	0.909	-0.018	0.982	0.068	0.787	-0.055	0.946	0.070	0.432	-0.097	0.907	0.064	0.129	-0.101	0.904	0.066	0.124	-0.117	0.890	0.063	0.067
**Job**																								
White Collar	Ref.				Ref.				Ref.				Ref.				Ref.				Ref.			
Pink Collar	0.193	1.213	0.091	0.034	0.209	1.233	0.098	0.034	0.217	1.243	0.095	0.024	0.095	1.100	0.077	0.218	0.095	1.099	0.077	0.222	0.090	1.094	0.077	0.243
Blue Collar	0.166	1.181	0.066	0.013	0.166	1.181	0.069	0.016	0.194	1.214	0.069	0.005	0.093	1.098	0.075	0.217	0.082	1.086	0.075	0.269	0.089	1.093	0.075	0.238
Unemployed	0.189	1.208	0.073	0.010	0.228	1.257	0.077	0.003	0.251	1.285	0.075	0.001	0.124	1.132	0.066	0.062	0.116	1.123	0.066	0.083	0.123	1.130	0.064	0.057
**Area**																								
Urban	Ref.				Ref.				Ref.				Ref.				Ref.				Ref.			
Rural	0.034	1.034	0.057	0.556	0.040	1.040	0.055	0.473	0.036	1.036	0.055	0.516	-0.089	0.915	0.058	0.127	-0.088	0.915	0.057	0.120	-0.083	0.920	0.056	0.139
**Sleep**																								
<7 hr	Ref.				Ref.				Ref.				Ref.				Ref.				Ref.			
> 7 hr	-0.060	0.942	0.052	0.256	-0.057	0.944	0.054	0.289	-0.073	0.930	0.054	0.177	-0.101	0.904	0.043	0.021	-0.104	0.901	0.044	0.020	-0.115	0.891	0.043	0.008
**Physical Activity**																								
No activity	Ref.				Ref.				Ref.				Ref.				Ref.				Ref.			
Walking	-0.067	0.935	0.074	0.364	-0.093	0.912	0.074	0.211	-0.087	0.917	0.074	0.240	-0.157	0.855	0.057	0.007	-0.163	0.850	0.057	0.005	-0.167	0.846	0.057	0.004
MVPA[a]	-0.094	0.911	0.138	0.497	-0.155	0.856	0.143	0.278	-0.164	0.849	0.147	0.264	-0.085	0.919	0.141	0.548	-0.075	0.928	0.142	0.600	-0.099	0.906	0.140	0.480
Walking+MVPA[a]	-0.223	0.800	0.079	0.005	-0.274	0.760	0.080	0.001	-0.279	0.757	0.081	0.001	-0.247	0.781	0.064	0.0001	-0.253	0.776	0.064	0.0001	-0.265	0.767	0.064	<.0001

Multiple regression analysis and linear trend results are shown.
* P-value for linear trend
** e^ β shows a percental increase of 10-year ASCVD risk score compared with the reference criteria
[a] MVPA: moderate to vigorous physical activity which includes vigorous intensity, moderate intensity, and strength-related physical activities
[b] ASCVD: atherosclerotic cardiovascular diseases

Table 3. Subgroup analysis of the effect of the blood heavy metal quartile

Variables			ASCVD[a] risk score (log-transformed)															
			Location of living								Sleep							
			Urban				Rural				under 7 hours				over 7 hours			
			β	e^β*	S.E	P-value	β	e^β*	S.E	P-value	β	e^β*	S.E	P-value	β	e^β*	S.E	P-value
Men		25%	ref.				ref.				ref.				ref.			
	Quartile Cd	50%	0.267	1.307	0.070	0.0001	-0.111	0.895	0.136	0.413	0.200	1.221	0.073	0.007	0.267	1.307	0.118	0.024
		75%	0.317	1.373	0.076	<.0001	-0.056	0.945	0.136	0.680	0.253	1.287	0.082	0.002	0.319	1.376	0.105	0.003
		100%	0.415	1.515	0.083	<.0001	0.215	1.240	0.142	0.132	0.360	1.433	0.088	<.0001	0.469	1.599	0.112	<.0001
		25%	ref.				ref.				ref.				ref.			
	Quartile Pb	50%	0.028	1.029	0.085	0.740	0.125	1.133	0.168	0.458	0.030	1.030	0.096	0.759	0.024	1.025	0.130	0.851
		75%	0.077	1.081	0.078	0.324	0.162	1.176	0.201	0.421	0.032	1.033	0.099	0.748	0.154	1.167	0.111	0.166
		100%	0.148	1.160	0.083	0.075	0.168	1.183	0.158	0.286	0.112	1.118	0.084	0.184	0.239	1.270	0.129	0.065
		25%	ref.				ref.				ref.				ref.			
	Quartile Hg	50%	0.082	1.085	0.078	0.298	-0.109	0.896	0.127	0.389	0.160	1.173	0.095	0.093	-0.031	0.969	0.112	0.780
		75%	0.170	1.185	0.086	0.048	0.023	1.023	0.132	0.863	0.251	1.285	0.090	0.005	0.042	1.043	0.121	0.730
		100%	0.175	1.191	0.090	0.053	-0.025	0.975	0.141	0.859	0.159	1.172	0.098	0.105	0.168	1.183	0.130	0.196
Women		25%	ref.				ref.				ref.				ref.			
	Quartile Cd	50%	0.103	1.108	0.063	0.104	-0.368	0.692	0.147	0.013	0.126	1.134	0.076	0.097	-0.097	0.908	0.093	0.301
		75%	-0.015	0.985	0.060	0.799	-0.006	0.994	0.114	0.958	0.041	1.042	0.069	0.552	-0.128	0.879	0.089	0.151
		100%	0.153	1.165	0.062	0.015	0.089	1.093	0.130	0.494	0.152	1.164	0.080	0.059	0.101	1.106	0.082	0.218

**Quartile Pb**	25%	**ref.**				**ref.**				**ref.**				**ref.**			
	50%	-0.002	0.998	0.068	0.974	0.013	1.013	0.118	0.911	0.066	1.068	0.076	0.382	-0.072	0.931	0.085	0.397
	75%	-0.012	0.988	0.061	0.842	0.061	1.063	0.125	0.624	0.072	1.074	0.068	0.296	-0.077	0.926	0.089	0.388
	100%	0.030	1.030	0.065	0.647	0.330	1.391	0.137	0.017	0.142	1.152	0.075	0.060	0.010	1.010	0.087	0.908
**Quartile Hg**	25%	**ref.**				**ref.**				**ref.**				**ref.**			
	50%	0.032	1.033	0.063	0.608	0.058	1.060	0.098	0.553	0.071	1.074	0.074	0.340	0.003	1.003	0.082	0.968
	75%	0.085	1.088	0.066	0.197	0.164	1.178	0.158	0.300	0.124	1.132	0.078	0.112	0.063	1.065	0.093	0.502
	100%	0.171	1.186	0.062	0.006	0.198	1.219	0.127	0.119	0.163	1.177	0.074	0.029	0.181	1.199	0.093	0.053

Multiple regression analysis results are shown.
* e^ β shows a percental increase of 10-year ASCVD risk score compared with the reference criteria
[a] ASCVD: atherosclerotic cardiovascular diseases
